# Infants are sensitive to the social signaling value of shared inefficient behaviors

**DOI:** 10.1038/s41598-023-46031-0

**Published:** 2023-11-16

**Authors:** Jesús Bas, Olivier Mascaro

**Affiliations:** 1 Center for Brain and Cognition, Universitat Pompeu Fabra, 08005 Barcelona, Spain; 2 Université Paris Cité, CNRS, Integrative Neuroscience and Cognition Center, 75006 Paris, France

**Keywords:** Psychology, Human behaviour

## Abstract

Actions that are blatantly inefficient to achieve non-social goals are often used to convey information about agents’ social affiliation, as in the case of rituals. We argue that when reproduced, actions that are individually inefficient acquire a social signaling value owing to the mechanisms that support humans’ intuitive analysis of actions. We tested our hypothesis on 15-month-old infants who were familiarized with an agent that reproduced or merely observed the actions of efficient and inefficient individuals. Subsequently, we measured the infants' expectations of the agent’s preferences for efficient and inefficient individuals. Our results confirmed that when agents act alone, infants expect a third-party to prefer efficient over inefficient agents. However, this pattern is entirely flipped if the third-party reproduces the agents’ actions. In that case, infants expect inefficient agents to be preferred over efficient ones. Thus, reproducing actions whose rational basis is elusive can serve a critical social signaling function, accounting for why such behaviors are pervasive in human groups.

## Introduction

From traditional rituals to modern viral internet challenges, activities that may appear puzzling from a causal or instrumental viewpoint are ubiquitous in human societies^1,2^. Scientists have long been fascinated by the factors that explain why these behaviors spread and remain culturally stable. Many researchers posit that these behaviors owe their cultural success to their capacity to act as powerful social signals^3–5^. We argue that such social signaling powers result from humans’ intuitive representations of actions and test this hypothesis among infants. We focus on actions that are inefficient to achieve non-social goals. From an observer’s viewpoint, these actions appear sub-optimal for achieving goals in the physical world, such as moving toward a location, or manipulating objects. Throughout the study, we refer to these actions as “inefficient actions”.

### The paradoxical social success of inefficient actions

Predicting and interpreting the behavior of others is an extremely complex task. One way to address this challenge is to assume that individuals behave rationally^6^, i.e., act to optimize their expected utility by minimizing costs and maximizing benefits^7,8^. Such expectations of instrumental rationality (or their functional equivalent) appear early in the development of humans. Infants expect others to act efficiently to reach their goals, for example, by using the shortest available route to reach a target rather than making unnecessary detours^9–14^. In short, from infancy onward, humans seem to predict and interpret others’ behaviors through expectations of instrumental rationality that support rich sets of structured inferences about people’s behaviors^7,10,15,16^.

Being sensitive to people’s rationality is not only important for predicting and interpreting their behavior, but it is also crucial for evaluating potential social partners. As cooperation is central to human social life, we are likely to be equipped with mechanisms for partner choice that support preferences for the best possible social partners^17,18^. In a partner choice context, constantly acting in an inefficient way without apparent reason might be negatively evaluated by third-party observers. First, behaviors that cannot be explained by appealing to expectations of efficiency are difficult to predict and interpret. They are cognitively opaque, in that it is difficult to determine why the agent performing them act the way they do^19^. Acting in a consistently opaque manner might be negatively evaluated, because it is hard to interact successfully with agents whose actions appear highly unpredictable and unintelligible. Second, inefficient behaviors can cue incompetence by assuming that the agent performing them acts in an unnecessarily costly manner. Thus, agents whose behaviors are systematically inefficient might be devalued social partners because they appear either wholly unpredictable, or incompetent.

Indeed, infants are more likely to learn labels from previously efficient rather than previously inefficient agents (Colomer & Woodward, 2023). Moreover, infants expect agents to prefer other efficient agents to inefficient ones. In Colomer et al.’s^20^ study, 15-month-old infants saw an observer watching two other characters perform goal-directed actions. One character acted efficiently, whereas the other did not. Subsequently, during the test phase, the infants expected the observer to preferentially approach the efficient rather than the inefficient character. Thus, from infancy onward, humans expect blatantly inefficient agents to be devalued social partners.

However, behaviors that are inefficient in achieving non-social goals also appear to act as powerful cues for bonding and for group cohesion between the individuals performing them^21–24^. For example, rituals convey a wealth of information about social bonds, even though the causal and instrumental relevance of the actions they involve is often hard to evaluate^21,22^. Even infants appear sensitive to the signaling value of behaviors whose nonsocial efficiency is elusive. They expect people to approach each other after seeing them move in the same manner in the absence of any identifiable external non-social goal^25–28^. Moreover, 16-month-old infants expect people performing the same causally and instrumentally mysterious action (lighting a lamp by pressing on it with one’s forehead) to be more likely to be affiliated with each other than people who perform different actions^29^.

In short, behaviors that are inefficient to achieve non-social goals raise a paradox. When performed by lone individuals, they can be negatively evaluated, because of their unpredictability, or because of their apparent inefficiency. However, individually inefficient behaviors that are performed by several individuals are often taken as cues of strong social bonds, such as during rituals^3–5^. This paradox can be solved by assuming that, when performed by several individuals, behaviors that are inappropriate for achieving nonsocial goals become powerful social signals.

### The social signaling value of reproducing inefficient actions

Behaviors that are blatantly inefficient in achieving non-social goals can gain a powerful social signaling value owing to the way humans spontaneously interpret actions. First, individuals are assumed to act in an efficient manner, thus inefficient behaviors are unexpected and consequently, *attention-grabbing*—a key feature of any signal. Indeed, from infancy onwards young humans dedicate more overt attention to inefficient than to efficient actions by looking longer at them^13,15,30^. More generally, many theories of cognition assume that detecting a mismatch between what was expected, and what is actually observed, drives learning^31,32^. Indeed, observing surprising events enhance infants’ learning^33–37^. Thus, the unexpectedness of inefficient behaviors makes them effective at attracting attention and at triggering learning.

Second, the motor reproduction of inefficient behaviors is likely to appear *intentional.* When a person’s movement is inefficient in achieving external physical goals, adults spontaneously rationalize that person’s behavior by assuming that producing the movement itself is the person’s goal^38^. Similarly, observing an individual performing the same inefficient actions as a model can trigger the inference that such individual aims to reproduce the motor behaviors of the model, which is a key property of genuine instances of imitation^39,40^.

Third, reproducing inefficient actions is *socially informative*. Constraints shape efficient actions. Two people may perform the same efficient action not because they share any special relation or knowledge but simply as a by-product of each person’s efficient adjustment to constraints. For instance, two people taking the shortest available route to reach a location may end up following the same path, not because one influences the other but simply because each of them is acting efficiently. In contrast, the space for possible actions whose features cannot be explained by observable constraints is extremely vast. Thus, it is highly improbable that two people perform the same inefficient action by coincidence. For instance, it is unlikely that two people would follow just by chance the exact same unnecessarily long and complicated path to reach a location. Consequently, the reproduction of inefficient actions is socially informative. It provides evidence that people either influence each other or share knowledge.

Fourth, performing inefficient actions is *costly*. Thus, observing a person reproducing the inefficient actions of a model provides a powerful demonstration of trust in the model. From infancy onward, humans infer the value of people’s goals from the costs of their actions; the higher the action cost, the higher the goal’s value^41^. Therefore, when a follower reproduces a model’s seemingly unnecessary and costly behaviors, observers may infer that the follower values reproducing the model’s behaviors, and that the model’s capacity to influence the follower is strong.

Fifth, actions that may appear blatantly inefficient in achieving nonsocial goals may be rationalized as *communicative*. Actions of this type efficiently convey that they are not directed toward the achievement of nonsocial goals. For instance, handwaving conspicuously does not achieve any non-social goal, but it might convey efficiently someone's intention to communicate. Accordingly, adults are more likely to assume that a behavior is communicative when it is unlikely to be produced pursuing a nonsocial goal^42^. To our knowledge, there is no comparable evidence showing that infants infer that behaviors inefficient to achieve non-social goals might be communicative. Yet, infants’ learning is enhanced when actions’ capacity to communicate information is increased at the expense of their non-social efficiency^43–46^. For instance, in a study by Hirai et al.^30^, 10-month-old infants observed an adult holding out an object. Infants memorized the object’s appearance specifically when the held-out gesture (i) followed an exaggerated trajectory that could not be explained by physical constraints, and (ii) was directed towards the infant. Thus, infants specifically learned about the object when the action could be construed as an instance of the adult showing the object to them communicatively^30^. In short, actions that are conspicuously inefficient to achieve non-social goals can serve a communicative function.

### Testing the hypothesis

In short, five key factors contribute to the social signaling power of inefficient behavior. The reproduction of these behaviors is likely to attract attention, and to be perceived as intentional, socially informative, costly, and communicative. Consequently, the reproduction of behaviors that are conspicuously inefficient in supporting non-social goals can act as a powerful signal of social bonds. To test this hypothesis we used a violation-of-expectation method^35,47^ that relies on infants’ tendency to look longer at events that they find unexpected or hard to process.

We capitalized on two different research areas. First, we built on studies showing that when agents act individually, 15-month-old infants expect agents to prefer efficient characters over inefficient ones^20^. Second, we draw on studies showing that infants expect individuals to affiliate with, follow and approach agents whose actions were reproduced by others^25–29^. We capitalize on these two sets of results to create an experiment in which we manipulated the efficiency of agents’ actions and whether these actions were merely observed or reproduced by a third-party.

If the inferences from efficiency and action reproduction were combined by adding their isolated effects, infants would expect the strongest third-party social preference for agents that are both (i) efficient and (ii) whose actions are reproduced. In contrast, our hypothesis predicted that infants would expect inefficient agents to be preferred over efficient agents when their behaviors are reproduced because of the social signaling value of shared inefficient action. Moreover, our hypothesis predicts that this pattern would be reversed when the agents’ behaviors are merely observed by a third-party agent.

**Figure 1:**
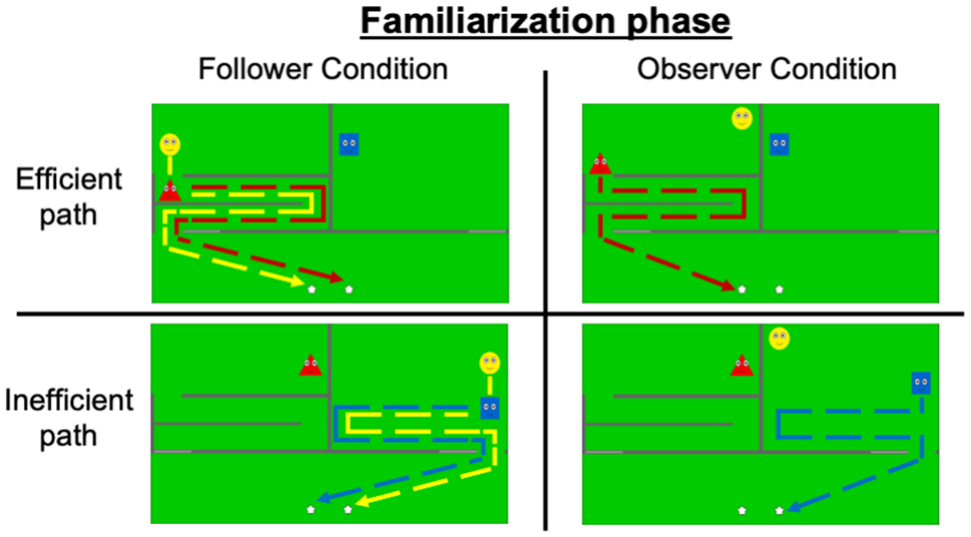
Experimental stimuli for the familiarization phase, per Condition (Follower vs. Observer) and familiarization movie type (efficient vs. inefficient). Colored dotted arrows represent the paths followed by the characters: they were not shown in the animations. Follower Condition: The follower (yellow) following an efficient (red) and an inefficient (blue) character. Note that the efficient and the inefficient characters followed paths of identical length and complexity. However, the complicated path followed by the efficient character was the shortest available route to reach the targets at the bottom of the screen. Conversely, the inefficient character could have taken a much shorter path to reach the targets. Observer Condition: The observer (yellow) observing an efficient (red) and an inefficient (blue) character.

**Figure 2:**
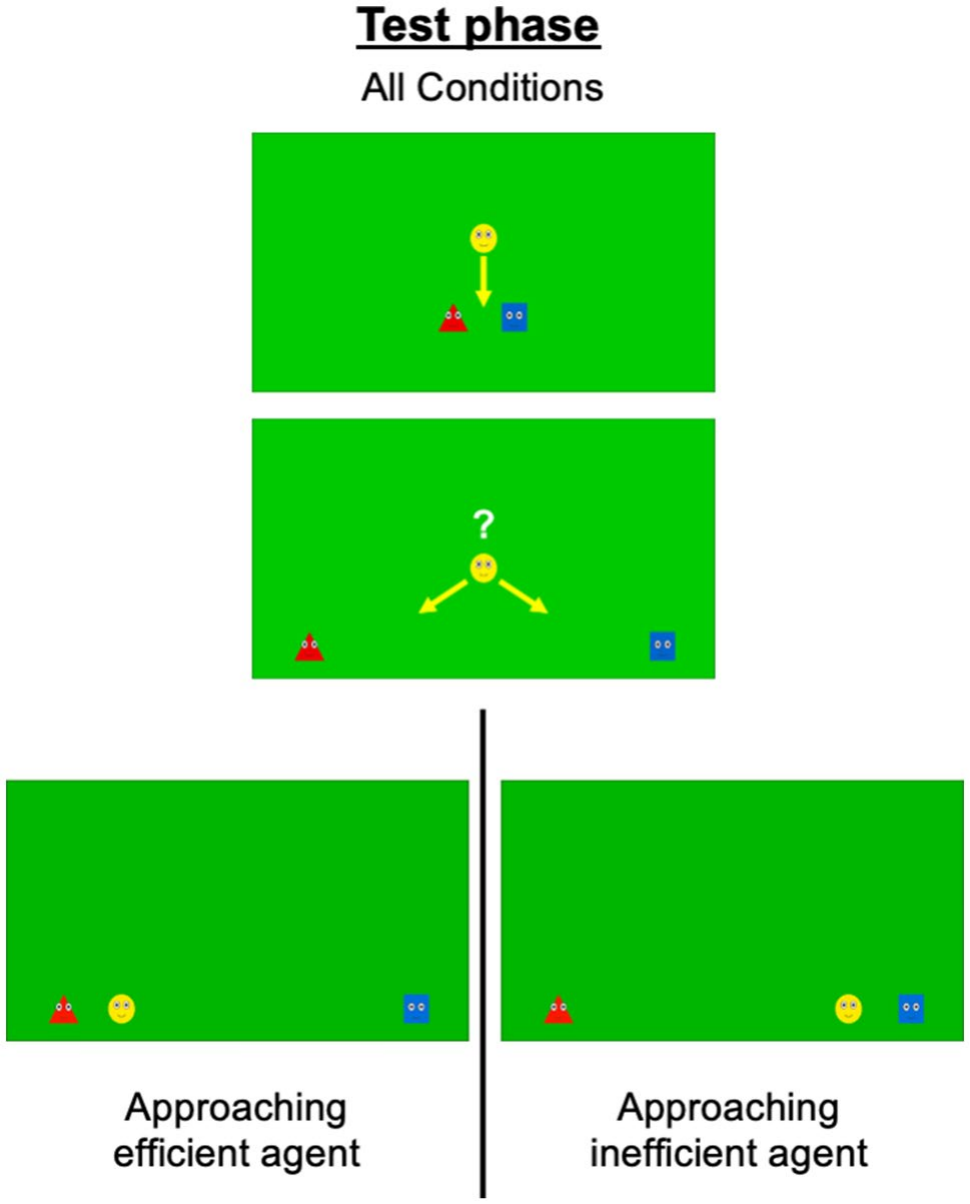
Experimental stimuli for the test phase (both Conditions). The follower (Follower condition) or the observer (Observer Condition) chooses between approaching and following either the efficient (red) or the inefficient (blue) character.

In the Follower Condition, 15-month-old infants were familiarized with videos in which a character (the follower) reproduced the actions of two other characters: one who acted efficiently (i.e., took the shortest available path to reach a target, Video S1), while the other acted inefficiently (i.e., took an unnecessarily long path while a much shorter route was available, Video S2, Fig. 1). Later, in the test phase, we measured the infants’ looking times when the follower followed and approached either the efficient or inefficient character (Video S3, Fig. 2). If infants treat the reproduction of inefficient actions as a stronger cue for social influence than efficient actions, they would expect a third party to follow the inefficient character during the test. Moreover, we also ran an Observer Condition to ensure that infants’ expectations were reversed when the characters’ actions were not reproduced. The Observer Condition was identical to the Follower Condition, except that one character simply observed the efficient and inefficient characters during the familiarization phase, but did not follow them (Videos S4 and S5). Based on previous research^20^, we assumed that if infants tracked the efficiency of the agents’ behaviors, they would expect the observer to follow and approach the efficient character rather than the inefficient one (see Figs. 1 and 2 for a visual summary of the study design and methods for a comprehensive description).

## Results

**Figure 3:**
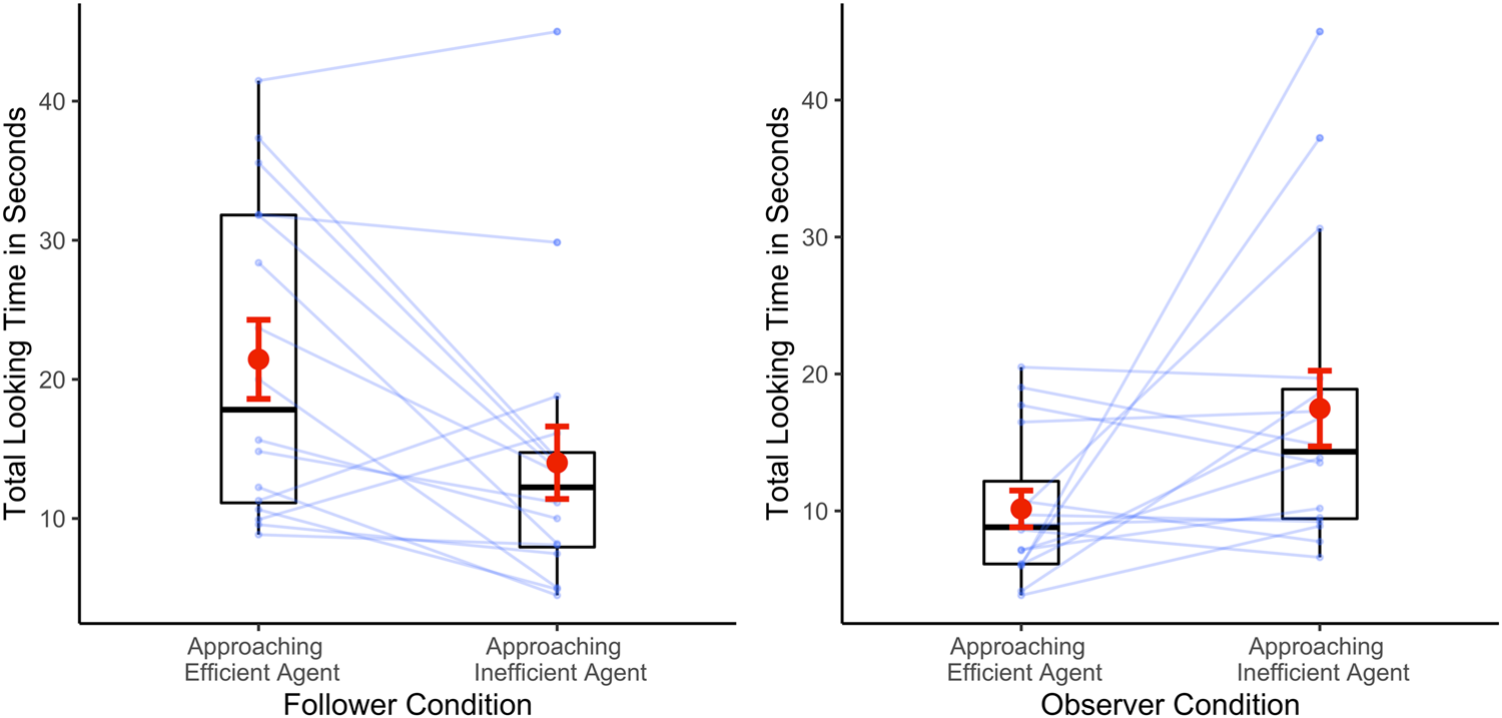
Infants' looking times at the screen (in seconds) per condition and type of test trial. Each pair of connected dots shows the data from each individual participant. Horizontal bars within boxes indicate medians, and boxes indicate the middle two quartiles of data. Upper whiskers indicate data up to 1.5 times the interquartile range above the third quartile, and lower whiskers indicate data up to 1.5 times the interquartile range below the first quartile. Red dots and error bars indicate means and SEMs.

The total screen-looking times of the participants during the test phase are shown in Fig. 3. We ran a mixed model ANOVA of the total looking time at the screen with condition (Follower vs. Observer) and test order (efficient first vs. inefficient outcomes first) as between-participants factors, and test trial type (approaching efficient agent vs. approaching inefficient agent) as a within-participants factor.

The ANOVA yielded only a statistically significant Interaction effect between condition and test trial type [*F* (1,28) = 16.257, *p* < 0.001, *η*^2^_p_ = 0.367]. Planned comparisons indicated that during the test phase of the Follower Condition, infants looked significantly longer when the character approached the efficient agent [*M* = 21.439 s, *SD* = 11.377 s, *95%* CI (15.382, 27.497)] rather than the inefficient agent [*M* = 14.008 s, *SD* = 10.422 s, *95%* CI (8.454, 19.562); *t*(15) = 3.373, *p* = 0.004, *d* = 0.843]. In the Observer Condition, this pattern was reversed; infants looked significantly longer when the character approached the inefficient agent [*M* = 17.475 s, *SD* = 11.057, *95%* CI (11.583, 23.367)] rather than the efficient agent [*M* = 10.149 s, *SD* = 5.363, *95%* CI (7.290, 13.007); *t*(15) = 2.615, *p* = 0.020, *d* = 0.654].

## Discussion

Many human behaviors may appear inefficient in achieving non-social goals. We suggested that the social signaling value of these behaviors contributes to the ease with which they spread; specifically, we hypothesized that the reproduction of actions that are inefficient in achieving non-social goals can be a powerful cue of social bonds.

To test our hypothesis, we familiarized infants with a follower reproducing (Follower Condition) or observing (Observer Condition) the actions of efficient and inefficient agents. In the test phase, we measured the infants’ looking times when the follower followed and approached either of the other two agents. In the Observer Condition, infants expected the follower to preferentially follow an efficient agent rather than an inefficient one—a pattern that dovetails with past results^20^. This result suggests that infants are sensitive to the efficiency of agents’ actions and, in line with theories of partner choice, infants expect agents to approach the most efficient partner. However, infants’ expectations of third-party preferences for efficient and inefficient agents changed drastically when their actions were reproduced. In the Follower Condition, the follower reproduced the paths of the inefficient and efficient agents. Subsequently, during the test phase, the infants expected the follower to preferentially follow and approach the inefficient agent rather than the efficient one in a new scenario. Thus, infants treated the reproduction of inefficient actions as a powerful social cue that could override their tendency to assume that inefficient agents may be devalued by social partners. As we argued, the strong social signaling powers of inefficient actions are likely to result from the structure of humans’ action understanding mechanisms.

These results have implications for understanding children’s processing of imitative behaviors. Young children and toddlers sometimes reproduce causal and unnecessary actions demonstrated by a model, a phenomenon sometimes called over-imitation^48^, blanket copying^49^, or indiscriminate imitation^50^. Several researchers have argued that children’s reproduction of such actions can be explained, at least in part, by socio-cognitive mechanisms and social motivations^51–53^. In line with these proposals, our data indicate that by 14 months of age, infants treat the reproduction of actions that are causally unnecessary to achieve a physical goal as a social cue relevant to inferring social bonds.

Discriminating between genuine and spurious instances of imitation is challenging. Our data suggest that from infancy onward, humans do more than track whether people perform the same movements when processing the behaviors of agents acting alike. Strikingly, during the familiarization phase of the Follower Condition, the followers acted as the efficient and the inefficient models in equal measures. However, the similarity between the actions of the follower and those of the efficient agent could be accounted for by the situational constraints. Conversely, there was no visible constraint explaining why the follower moved along the same path as the inefficient agent in the Follower Condition, thus providing infants with stronger evidence for intentional imitation. Therefore, when identifying imitative behaviors, infants do not just register whether people act in the same manner. They also consider whether situational constraints could account for behavioral similarities.

These results dovetail with studies on the capacity to track the social transmission of artefacts’ designs. Adults and preschoolers assume that identical tools are less likely to be copies when their similarity can be explained by functional constraints, rather than when it cannot^54–56^. For instance, in a set of studies, participants had to determine whether an individual copied another one when building train tracks. Adults, and 7- to 9-year-old children judged identical efficient tracks as less likely to be copies than identical inefficient tracks^54,56^. Together with our data, these results indicate that from an early age, humans go beyond applying simple rules based on tracking the similarity between actions or designs (e.g., “acting alike = imitating” or “acting alike = being affiliated”). Instead, infants use information about the cause of similarities to adjust their social inferences, and they discount similarities that can be explained by external constraints.

Our results indicate that infants react to behaviors that are inefficient to achieve non-social goals. However, we do not claim that infants directly represent and evaluate people’s efficiency in our study. While this is a possibility, infants may also react to correlates of inefficiency, such as the predictability of people’s behaviors. In our study, the path taken by the efficient agent is predictable, and it can be explained by the constraints created by the maze. In contrast, the behavior of the inefficient agent is hard to predict, and opaque—it is it hard to determine exactly why the agent is taking a convoluted, complex path. Thus, in our study, infants might simply encode that the inefficient agent is acting in an unpredictable manner, without necessarily evaluating this agent as inefficient or incompetent per se.

Our study has several limitations that offer possibilities for future research. First, we showed that the reproduction of inefficient actions is more informative about people’s social bonds than the reproduction of efficient actions. However, we did not investigate the type of social inference that infants might draw from observing the reproduction of inefficient actions in detail. For instance, it would be interesting to test whether infants assume that people whose inefficient actions are reproduced have more status or knowledge than their followers. Second, we did not assess the inferences that infants might draw about reproducing inefficient actions. It is often assumed that the propensity to reproduce causally opaque actions can serve as a vehicle for the transmission of norms and culturally shared knowledge^57^. Thus, future studies might investigate whether infants are more likely to interpret an apparently inefficient action as (i) resulting from a piece of shared cultural knowledge and (ii) in a normative manner. Third**,** we focused on one specific situation, namely path-following. In future studies, it would be important to test whether the type of expectations that we observed generalizes to a broader set of contexts, such as tool-use for instance**.** Fourth and lastly, our sample sizes were set a priori using a power analysis, and our interpretations are based on a clear pattern of statistically significant results. Yet, as in many comparable studies^25,27–29^, our sample sizes are relatively small (*n* = 16 per group), and we tested participants from a single area (large French cities). Replicating our results with a larger set of participants from a wider variety of backgrounds would be important to probe further the generalizability of our findings.

In conclusion, we showed that from infancy onward, actions whose non-social rationality is elusive can serve a critical social signaling function by building minimally on humans’ intuitive analysis of actions. This result contributes to explain why behaviors that might appear irrational at first glance can spread and remain stable in human groups.

## Methods

### Ethical approval

The study was conducted in accordance with the principles of the Declaration of Helsinki and was approved by an independent ethical review committee (Comité de Protection des Personnes Sud-Est II Auvergne-Rhône Alpes, IRB: 00009118). All parents signed informed consent forms for their infants to participate in this study.

### Participants

Two groups of 15-month-old infants were included in the analysis (Follower Condition: *n* = 16; M = 474, SD = 9 days; 10 males; Observer Condition: n = 16; M = 470, SD = 11 days; 7 males). We chose to test infants at 15 months because this is the youngest age at which the ability to infer social preferences based on agents’ efficiency has been evidenced^20^. Following the current standards in infancy research, we conducted a power analysis to set the sample size a priori using G*Power (v3.1; Faul et al.^58^). With α = 0.05 and assuming an effect size equal to the one observed in Colomer et al.’s study (d = 0.849)^20^, a sample size of 14 was sufficient to achieve a power equal to 0.9 for within group comparisons of average looking times with t-tests for matched pairs. We increased the number of participants to 16 to counterbalance low-level factors and to fit the sample size recommended for infants’ total looking time studies^59^.

Thirteen infants were tested but were excluded from the final analysis. The exclusion criteria were crying or refusing to complete the experiment (5), moving in a way that prevented proper coding (3), parental interference (2), or experimental error (3). The participants were recruited by sending letters to a large sample of randomly selected infants born in the Lyon area (France). All participants were healthy full-term infants (≥ 37 weeks of gestation).

### Apparatus

The participants were tested in a sound-attenuated room. The infants sat on their caregivers’ lap approximately 65 cm from a 23″ screen (resolution 1920 × 1080 pixels) on which the stimuli were presented. The participants’ behavior during the session were recorded using a Sony HDR-HC9E camera (temporal resolution: 50 frames/s). The stimuli were presented using PsychoPy (v.3.0).

### Stimuli

The stimuli were computer-based animations involving three different “agents” represented by abstract geometric figures, each with a pair of eyes. All videos were created using the Adobe Flash Animation software.

#### Follower condition

##### Familiarization phase

In the Follower Condition, the apparent efficiency of the agents’ actions was manipulated. Fifteen-month-old infants were familiarized with videos in which a “follower” followed two other agents: one who acted efficiently (i.e., took the shortest available path to reach a target), while the other acted inefficiently (i.e., took an unnecessarily long path while a much shorter route was available).

The way objects move, individually and in relation to each other, influence infants’ attention from an early age. Before their first birthday, infants are sensitive to motion properties such as speed and trajectory^60,61^, and they discriminate agents chasing or following each other from agents moving independently^62–64^. Thus, in our study, we controlled for the influence of agents’ movements by keeping them constant across types of familiarization trials (efficient vs. inefficient). To manipulate agents’ efficiency while keeping their movements identical, we modified the constraints that conditioned agents’ movements^30,42,55,56^.

During each familiarization trial in the Follower Condition, the infants saw a video showing the follower following a second agent along a circuitous path to reach a target (small stars). In the efficient familiarization trials, the follower had no option but to move along a circuitous path to reach the target because their movements were constrained by the walls of a maze (Fig. 1, top-left picture). In the inefficient familiarization trials, the follower followed an equally convoluted route. However, there was no maze. Thus, in the inefficient familiarization trials, the agents took an unnecessarily long path to reach the target even though a much shorter option was available (Fig. 1, bottom-left picture).

In each familiarization movie, two rectangular areas of identical size and shape were demarcated, one on each side of the screen's top half: an enclosure with a maze and an empty enclosure. Inside the maze enclosure, walls created a circuitous labyrinth that connected to the bottom half of the screen through an exit door (Fig. 1, upper-left of the pictures). The empty enclosure was identical, except it contained no labyrinth (Fig. 1, upper-right of the pictures). Each familiarization movie included three characters: an efficient agent, an inefficient agent, and a follower.

At the beginning of the efficient familiarization trials, the efficient agent and the follower were located at the top of the maze enclosure. The inefficient agent was at the top of the empty enclosure. The exit door at the bottom of the maze was open, whereas that at the bottom of the empty enclosure was closed. This setup was displayed for 1 s until two stars appeared at the bottom of the screen while a sound was played (1.33 s). The efficient agent then moved toward the entrance of the maze (depending on the maze's shape, this segment lasted 0.66, 1.33 or 2.66 s) and “called” the follower by emitting a sound while rocking gently from left to right (1 s). The follower approached the agent (0.4, 1.8, or 2.9 s) and subsequently followed them through the maze until they reached the exit at the bottom of the screen (9.4, 9.5, or 9.7 s). Once the efficient agent and follower reached the exit, each made contact with one of the stars (4.2 s) and jumped twice while a sound was played (2.5 s). Next, the movie froze, and the image remained on the screen for 1 s (Video S1).

The inefficient familiarization trials were exactly the same as the efficient familiarization trials, with only three differences: (1) at the beginning of the familiarization trials, instead of being located next to the efficient agent, the follower was inside the empty enclosure next to the inefficient agent; (2) the exit at the bottom of the maze was closed, whereas the exit at the bottom of the empty enclosure was opened; and (3) the movements of the agents were the same as in the efficient familiarization trials. Crucially, there were no walls inside the empty enclosure. Thus, from a third-party perspective, the inefficient agent and follower took an unnecessarily circuitous path to reach the exit and access the stars (Video S2).

**Figure 4:**
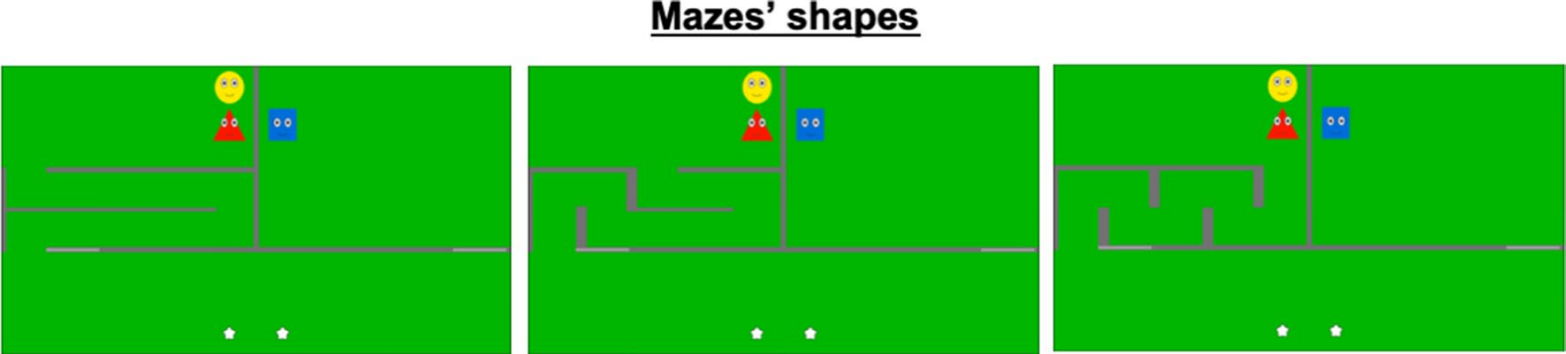
Mazes’ shapes. During familiarization, agents’ paths were determined by three different labyrinths. Each of them was used for one efficient familiarization movie, and for one inefficient familiarization movie.

Three efficient familiarization trials were interspersed with three inefficient familiarization trials (starting with an efficient familiarization trial for half the participants and using the opposite pattern for the other half). Between each trial, a fixation cross was presented for 0.5 s at the center of a gray screen. Three different geometrical shapes were used to represent the agents. For half of the participants, the efficient and inefficient agents were represented by a red triangle and a blue square, respectively. We used the opposite pattern for the other half of the participants. The follower always appeared as a yellow circle. During the familiarization phase, the side of the screen where the maze enclosure was located remained constant for each individual participant. It was counterbalanced across participants. To alleviate infants’ boredom and help them generalize their expectations across situations, we used three different labyrinths inside the maze area—one per efficient familiarization trial (Fig. 4). The agents’ movements varied according to the shape of each labyrinth. In both efficient and inefficient familiarization movies, the two moving agents followed the same tortuous path (whose shape matched that of the labyrinth) and, in the end, each one of them collected a star. Each inefficient familiarization movies matched one of the efficient familiarization movies in that it used the same labyrinth shape. Yet, in the inefficient familiarization movies, the agents moving through the empty enclosure followed the tortuous path corresponding to the labyrinth shape, although a shorter path was available. Two trials using the same labyrinth shape were never presented consecutively. Familiarization lasted for 130 s, was followed by two consecutive test trials.

##### Test phase

During the test phase, we measured the infants’ looking time when the follower followed and approached either the efficient or inefficient agent. During each test trial, the participants saw a movie starting with a pre-test phase, followed by a test phase. At the beginning of the pre-test phase, the follower was located above the efficient and inefficient agents in the top part of the empty area (1 s). Then, the efficient and the inefficient agents slid down along parallel zigzagging paths (1 s), before “calling” the follower by emitting a sound while rocking gently from left to right (0.5 s). The follower then moved toward them (1 s). This sequence of movements (efficient and inefficient agents moving downward before being followed by the follower) was repeated four times (the agents slid down diagonally left and right twice each). At the end of the pre-test phase (11 s), the agents ended up in the center of the screen.

Subsequently, the test phase started. The efficient and inefficient agents slid down toward the bottom of the screen, first together (1 s) and then in opposite directions (one toward the bottom left corner, and the other toward the bottom right corner of the screen) (2 s). Next, they simultaneously “called” the follower by emitting a sound while rocking gently from left to right (0.5 s). The follower then slid down along the central vertical axis (1 s), paused (0.5 s), and followed the path of the efficient (efficient outcome) or inefficient (inefficient outcome) (1.5 s) agent. Once the follower arrived next to one of the two other agents, the video froze until the infant looked away from the screen for 2 s or after 45 s had elapsed. Each infant was presented with one efficient and one inefficient outcome test trial (Video S3).

We counterbalanced the following factors across participants: the agent that acted first during the familiarization phase (efficient or inefficient agent); the location of the maze on the screen during the familiarization phase (right or left side of the screen); the location of the efficient and inefficient agents on the screen during the familiarization and the test phase (efficient agent on the right side and inefficient agent on the left side, or the reverse pattern); and the order of presentation of test trials (efficient outcome first or inefficient outcome first).

#### Observer condition

The Observer Condition followed the same procedure as the Follower Condition, except that during the familiarization trials the follower did not follow anyone and remained motionless (Fig. 1, upper-right and bottom right pictures; Videos S4 and S5).

### Coding and analysis

The total time the participants spent looking at the screen was calculated online. During the test phase, the experimenter pressed a button when the infants looked at the screen and stopped pressing the button when they looked away. The test phase ended when PsychoPy (v3.0) calculated that infants were looking away for 2 s or after 45 s had elapsed from the beginning of the measurement period. At the end of the experimental session, PsychoPy (v3.0) provided the total looking time for each test phase. All analyses were performed using the R software (v.4.1.0), using the following packages: readxl (v.1.4.2), tidyverse (v.2.0.0), rstatix (v.0.7.0), patchwork (v.1.1.3).

An independent coder, who was unaware of the study hypothesis, coded half of the data. They analyzed whether the infants looked at the screen or looked away during the test phases frame-by-frame, using the same criteria as those used for online coding. Blinks were considered as looking away if they lasted for more than 0.2 s. Because a high inter-coder agreement was achieved between the online and offline data (ICC = 0.996, p < 0.01), we used the data from the online coding to perform our analyses.

Shapiro–Wilk tests revealed that the looking times departed from the normal distribution in the two test phases of both studies (Follower Condition: efficient test outcome: *W* = 0.885, *p* = 0.046; inefficient test outcome: *W* = 0.776, *p* = 0.001; Observer Condition: efficient test outcome: *W* = 0.874, *p* = 0.032; inefficient test outcome: *W* = 0.823 *p* = 0.006). To better approximate the normal distribution, we log-transformed the raw data before performing parametric statistics^59^. For ease of reading, the untransformed raw data are depicted in Fig. 3 and reported in the manuscript (data for all participants can be found in the Supplementary Information file). The data used for the main analysis (infants' looking times at the screen obtained by online coding) are also available in the Supplementary Information, along with the analysis script. All statistical tests reported in this manuscript were two-tailed.

### Supplementary Information

Supplementary Video S1.

Supplementary Video S2.

Supplementary Video S3.

Supplementary Video S4.

Supplementary Video S5.

Supplementary Information 1.

Supplementary Information 2.

## Data Availability

All data generated and analyzed during this study—infants' looking times at the screen obtained by the online coding—are included in this published article and its supplementary information files.
