# Supplementary material for: Infants are sensitive to the social signaling value of shared inefficient behaviors
Source: Sci Rep. 2023 Nov 16;13:20034. doi: 10.1038/s41598-023-46031-0 (PMC10654565; doi:10.1038/s41598-023-46031-0)
Supplement: Supplementary file 6 — Supplementary Information 1. [file 41598_2023_46031_MOESM6_ESM.pdf]

```
#### Analyses Script: Infants are sensitive to the social signaling value
of shared inefficient behaviors

### Preparing the working environment
## Load packages
library(readxl)
library(tidyverse)
library(rstatix)
library(patchwork)

## Load and select data for the main analyses
data <-
read_excel("Supplementary_Information_Infants_sensitivity_to_social_signaling_value_of_[illegible]
sheet = "Individual_Data")
data_1 <- data %>%
  select("Subject", "Condition","Efficient_Outcome", "Inefficient_Outcome",
"Test_video_1") %>%
  rename(Order_Test = Test_video_1)

### Prepare Data
## Check normality with Shapiro-Wilk Test for Normality
# Shapiro-Wilk - Follower Condition - Efficient_Outcome
sw_test_Follower_Efficient_Outcome <- data_1 %>%
  filter(Condition == "Follower") %>%
  pull(Efficient_Outcome) %>%
  shapiro.test()
sw_test_Follower_Efficient_Outcome

# Shapiro-Wilk - Follower Condition - Inefficient_Outcome
sw_test_Follower_Inefficient_Outcome <- data_1 %>%
  filter(Condition == "Follower") %>%
  pull(Inefficient_Outcome) %>%
  shapiro.test()
sw_test_Follower_Inefficient_Outcome

# Shapiro-Wilk - Observer Condition - Efficient_Outcome
sw_test_Observer_Efficient_Outcome <- data_1 %>%
  filter(Condition == "Observer") %>%
  pull(Efficient_Outcome) %>%
  shapiro.test()
sw_test_Observer_Efficient_Outcome

# Shapiro-Wilk - Observer Condition - Inefficient_Outcome
sw_test_Observer_Inefficient_Outcome <- data_1 %>%
  filter(Condition == "Observer") %>%
  pull(Inefficient_Outcome) %>%
  shapiro.test()
sw_test_Observer_Inefficient_Outcome

## Data is skewed, as expected (cf. Csibra et al., 2016)
## Log-transform data for analyses
data_1 <- data_1 %>%
  mutate(
```

```
    Log_Efficient_Outcome = log10(Efficient_Outcome),
    Log_Inefficient_Outcome = log10(Inefficient_Outcome)
  )

## Checking that log transformation reduced skewness
# Shapiro-Wilk - Follower Condition - Efficient_Outcome
sw_test_Follower_Efficient_Outcome <- data_1 %>%
  filter(Condition == "Follower") %>%
  pull(Log_Efficient_Outcome) %>%
  shapiro.test()
sw_test_Follower_Efficient_Outcome

# Shapiro-Wilk - Follower Condition - Inefficient_Outcome
sw_test_Follower_Inefficient_Outcome <- data_1 %>%
  filter(Condition == "Follower") %>%
  pull(Log_Inefficient_Outcome) %>%
  shapiro.test()
sw_test_Follower_Inefficient_Outcome

# Shapiro-Wilk - Observer Condition - Efficient_Outcome
sw_test_Observer_Efficient_Outcome <- data_1 %>%
  filter(Condition == "Observer") %>%
  pull(Log_Efficient_Outcome) %>%
  shapiro.test()
sw_test_Observer_Efficient_Outcome

# Shapiro-Wilk - Observer Condition - Inefficient_Outcome
sw_test_Observer_Inefficient_Outcome <- data_1 %>%
  filter(Condition == "Observer") %>%
  pull(Log_Inefficient_Outcome) %>%
  shapiro.test()
sw_test_Observer_Inefficient_Outcome

### Main Analyses
# Prepare dataframe for analysis
data_2 <- data_1 %>%
  gather(key = 'Test_trial_type', value = 'Log_TLT',
'Log_Efficient_Outcome':'Log_Inefficient_Outcome')

# Perform ANOVA
anova_data_2 <- anova_test(
  data = data_2,
  dv = Log_TLT,
  wid = Subject,
  between = c(Condition, Order_Test),
  within = Test_trial_type,
  effect.size = 'pes'
)
anova_data_2

# Paired t-test for Follower Condition
ttest_follower_condition <- data_2 %>%
  filter(Condition == "Follower") %>%
```

```
  t_test(Log_TLT ~ Test_trial_type, paired = TRUE) %>%
  add_significance() %>%
  as.data.frame()
ttest_follower_condition

# Calculate Cohen's d for Follower Condition
dcohen_follower_condition <- data_2 %>%
  filter(Condition == "Follower") %>%
  cohens_d(Log_TLT ~ Test_trial_type, paired = TRUE) %>%
  as.data.frame()
dcohen_follower_condition

# Paired t-test for Observer Condition
ttest_Observer_condition <- data_2 %>%
  filter(Condition == "Observer") %>%
  t_test(Log_TLT ~ Test_trial_type, paired = TRUE) %>%
  add_significance() %>%
  as.data.frame()
ttest_Observer_condition

# Calculate Cohen's d for Observer Condition
dcohen_observer_condition <- data_2 %>%
  filter(Condition == "Observer") %>%
  cohens_d(Log_TLT ~ Test_trial_type, paired = TRUE) %>%
  as.data.frame()
dcohen_observer_condition

# Descriptive statistics for Efficient and Inefficient Outcome in the
Follower and Observer Conditions
# Create a function to calculate the descriptive data
calculate_descriptive_data <- function(data, alpha = 0.05) {
  n <- length(data)
  mean_data <- mean(data)
  sd_data <- sd(data)
  margin <- qt(1 - alpha / 2, df = n - 1) * (sd_data / sqrt(n))
  lower_ci <- mean_data - margin
  upper_ci <- mean_data + margin
  return(list(
    Mean = mean_data,
    SD = sd_data,
    Lower_CI = lower_ci,
    Upper_CI = upper_ci
  ))
}

# Descriptive data - Follower Condition - Efficient_Outcome
Efficient_Outcome_Follower_Condition <- data_1 %>%
  filter(Condition == "Follower") %>%
  pull(Efficient_Outcome)

Descriptive_Efficient_Outcome_Follower_Condition <-
calculate_descriptive_data(Efficient_Outcome_Follower_Condition, alpha =
0.05)
```

```
Descriptive_Efficient_Outcome_Follower_Condition

# Descriptive data - Follower Condition - Inefficient_Outcome
Inefficient_Outcome_Follower_Condition <- data_1 %>%
  filter(Condition == "Follower") %>%
  pull(Inefficient_Outcome)

Descriptive_Inefficient_Outcome_Follower_Condition <-
calculate_descriptive_data(Inefficient_Outcome_Follower_Condition, alpha =
0.05)
Descriptive_Inefficient_Outcome_Follower_Condition

# Descriptive data - Observer Condition - Efficient_Outcome
Efficient_Outcome_Observer_Condition <- data_1 %>%
  filter(Condition == "Observer") %>%
  pull(Efficient_Outcome)

Descriptive_Efficient_Outcome_Observer_Condition <-
calculate_descriptive_data(Efficient_Outcome_Observer_Condition, alpha =
0.05)
Descriptive_Efficient_Outcome_Observer_Condition

# Descriptive data - Observer Condition - Inefficient_Outcome
Inefficient_Outcome_Observer_Condition <- data_1 %>%
  filter(Condition == "Observer") %>%
  pull(Inefficient_Outcome)

Descriptive_Inefficient_Outcome_Observer_Condition <-
calculate_descriptive_data(Inefficient_Outcome_Observer_Condition, alpha =
0.05)
Descriptive_Inefficient_Outcome_Observer_Condition

### Plot (Figure 2)
# Prepare data for the plot
data_plot_follower_condition <- data_1 %>% filter (Condition == 'Follower')
%>%
  gather (key ='Test_trial_type',value= 'TLT',
'Efficient_Outcome':'Inefficient_Outcome')

data_plot_observer_condition <- data_1 %>% filter (Condition == 'Observer')
%>%
  gather (key ='Test_trial_type',value= 'TLT',
'Efficient_Outcome':'Inefficient_Outcome')

# Create plot - Follower Condition
plot_follower_condition <- ggplot
(data=data_plot_follower_condition,aes(y=TLT, x = Test_trial_type)) +
  geom_boxplot(color = 'black', width=0.25, outlier.colour = 'royalblue1',
outlier.alpha = 0.3, outlier.size = 0.7) +
  geom_point(color = 'royalblue1', alpha = 0.3, size = 0.7) +
  geom_line(aes(group = Subject), color = 'royalblue1', alpha = 0.3) +
  stat_summary(fun=mean, geom="point", size=3, color="red2", fill="red2") +
```

```
  stat_summary(fun.data = mean_se, geom = "errorbar", colour="red2", width
= 0.08, size = 1) +
  ylab("Total Looking Time in Seconds") +
  xlab("Follower Condition") +
  theme_classic() +
  scale_x_discrete(labels = c("Approaching \n Efficient Agent",
"Approaching \n Inefficient Agent"))

plot_follower_condition

# Create plot - Observer Condition
plot_observer_condition <- ggplot
(data=data_plot_observer_condition,aes(y=TLT, x = Test_trial_type)) +
  geom_boxplot(color = 'black', width=0.25, outlier.colour = 'royalblue1',
outlier.alpha = 0.3, outlier.size = 0.7) +
  geom_point(color = 'royalblue1', alpha = 0.3, size = 0.7) +
  geom_line(aes(group = Subject), color = 'royalblue1', alpha = 0.3) +
  stat_summary(fun=mean, geom="point", size=3, color="red2", fill="red2") +
  stat_summary(fun.data = mean_se, geom = "errorbar", colour="red2",
position = position_dodge(width = 5), width = 0.08, size = 1) +
  ylab("Total Looking Time in Seconds") +
  xlab("Observer Condition") +
  theme_classic() +
  scale_x_discrete(labels = c("Approaching \n Efficient Agent",
"Approaching \n Inefficient Agent"))

plot_observer_condition

# Combine and save plots
combined_plot <- (plot_follower_condition + plot_observer_condition)
combined_plot

ggsave(combined_plot, device = "png" ,filename="Figure_3.png",width = 20,
height=10, units = "cm", dpi = 900)
```
